# Evaluation of LoRa Technology in Flooding Prevention Scenarios

**DOI:** 10.3390/s20144034

**Published:** 2020-07-20

**Authors:** José Cecílio, Pedro M. Ferreira, António Casimiro

**Affiliations:** LASIGE, Faculdade de Ciências, Universidade de Lisboa, 1749-016 Lisboa, Portugal; pmf@ciencias.ulisboa.pt (P.M.F.); casim@ciencias.ulisboa.pt (A.C.)

**Keywords:** LoRa technology, flood prevention, performance evaluation

## Abstract

Global climate change originates frequent floods that may cause severe damage, justifying the need for real-time remote monitoring and alerting systems. Several works deal with LoRa (Long Range) communications over land and in the presence of obstacles, but little is known about LoRa communication reliability over water, as it may happen in real flooding scenarios. One aspect that is known to influence the communication quality is the height at which nodes are placed. However, its impact in water environments is unknown. This is an important aspect that may influence the location of sensor nodes and the network topology. To fill this gap, we conducted several experiments using a real LoRa deployment to evaluate several features related to data communication. We considered two deployment scenarios corresponding to countryside and estuary environments. The nodes were placed at low heights, communicating, respectively, over the ground and over the water. Measurements for packet loss, received signal strength indicator (RSSI), signal-to-noise ratio (SNR) and round-trip time (RTT) were collected during a period of several weeks. Results for both scenarios are presented and compared in this paper. One important conclusion is that the communication distance and reliability are significantly affected by tides when the communication is done over the water and nodes are placed at low heights. Based on the RTT measurements and on the characteristics of the hardware, we also derive a battery lifetime estimation model that may be helpful for the definition of an adequate maintenance plan.

## 1. Introduction

The rapid progress of the economy associated with a number of human activities is destroying the environment [1,2]. Each year, natural and human originated disasters are causing the global climate change, leading to infrastructural damages, economical crisis and distresses for the population. Flooding is one of the major disasters occurring in the world. Recent studies [3,4] indicate that the risk of flooding in Europe will increase in the near future. Despite the existence of satellite images systems that allow forecasting rainfall, there is a need for real-time monitoring and alerting systems to constantly monitor flow, precipitation level and water level, to make a reasonable decision on the necessary actions to prevent flooding. Monitoring the water level in rivers, bays and the sea is hence an important problem that has increasingly attracted the attention of the research community. Advances in wireless technologies and communication protocols have enabled the development of environmental monitoring systems based on low-cost, low-power and multi-functional sensors that, typically, are able to communicate within specific distance limits. Technologies such as Wi-Fi or Zigbee [5] are commonly used in monitoring setups. Wi-Fi offers high data rates (above the required for most sensor deployments), but the transmission range is relatively small and the power consumption is high, due to the need of processing heavy communication protocols. Zigbee offers adequate data rates and low energy consumption, but only works for short communication ranges. Alternatively, Global System for Mobile (GSM) [6] or satellite communications [7] can be used for long communication ranges, supporting data rates appropriate for most sensor deployments. However, these technologies are rarely employed in these applications due to their cost.

As an alternative to all the previously mentioned technologies, Low Power Wide Area Networks (LPWANs) have emerged as one of the most promising IoT enabled networking technologies due to an advantageous combination of features [8]: reduced use of bandwidth, capability to connect a huge number of devices, long-range links, low-cost devices, and energy efficiency. The majority of these features are common to the different LPWAN solutions [9]. In this work, the focus is on LoRa [10], a LPWAN low-cost technology that provides better communication range in comparison to other technologies due to its radio sensitivity [11]. In addition, LoRa power consumption is very low, offering extended battery-powered operating time.

In this paper, we study LoRa and evaluate its performance for environmental monitoring applications, particularly in flood prevention. Therefore, we consider nodes that are located near water areas, placed at low heights, and that communicate over the water. The Okumura-Hata propagation model [12] and the two-ray ground reflected model [13] are analyzed in terms of communication range versus node and gateway heights. Forecasts based on these models are compared with experimental results collected from real deployments. Two deployment scenarios are considered for comparison purposes: an estuary monitoring scenario, where communication is done over water, and a countryside environment monitoring scenario, where communication takes place over solid ground. In both cases, nodes are always placed at low heights in relation to the water and the ground.

Existing propagation models ([14,15] and others) take into account node and gateway heights. However, a vast majority of the works reported in the literature (e.g., References [16,17]), in which several scenarios have been considered to evaluate LoRa, mainly consider nodes placed at high heights in order to achieve large communication ranges. This motivated our experimental study to determine the reliability of LoRa networks in a real flood monitoring system, where nodes are placed at low heights and in which the communication link is established over the land or over the water.

The experimental work and the corresponding results and discussion are provided in this paper. In particular, we study the conditions under which reliable LoRa communication can be achieved when nodes are placed at low heights. Moreover, as the battery lifetime of a node is an important aspect when remote environmental monitoring applications are deployed, we also present a battery lifetime estimation approach for LoRa-based applications.

The rest of the paper is organized as follows: Section 2 discusses related work concerning the evaluation of LoRa technology in real application scenarios. The main characteristics of LoRa are presented in Section 3, while a theoretical analysis of over-the-air propagation models is presented in Section 4. Our experimental setup and the configurations used to evaluate the LoRa technology are described in Section 5. Section 6 discusses the importance of the first Fresnel zone clearance to the transmission quality, while Section 7 provides the obtained results and their analysis. Since the network lifetime is an important aspect for maintenance, mainly for batteries replacement, Section 8 presents the lifetime estimation approach for the battery of a LoRa node. Finally, conclusions and remarks are given in Section 9.

## 2. Related Works

The LoRa technology [10] has been attracting attention from the academic and industrial communities, with several studies on the different aspects of the technology, such as communication range, interference, scalability or energy efficiency. LoRa uses Chrip Spread Spectrum (CSS) modulation [18], which allows the configuration of the bandwidth, spreading factor, coding rate and transmission power. Due to its flexibility, it shows huge potential for numerous applications, including remote sensing applications. However, to the best of our knowledge, practical reports on the reliability of LoRa when communication is done over water surfaces, as in estuary monitoring scenarios, are still scarce.

Since the number of LoRa deployments are increasing for both urban and sub-urban areas, LoRa nodes will be exposed to high interference. One important characteristic of LoRa is its robustness against interference. Bor et al. present a performance analysis of a LoRa transceiver [19], demonstrating how concurrent non-destructive transmissions and carrier detection can be employed to extend the battery lifetime of nodes. The same authors also performed an experimental study [20] on the impact of configurable LoRa transmission parameters on the communication performance.

The physical and data link layer performance of LoRa have also been evaluated [21,22,23]. It has been shown that even in high interference scenarios, LoRa is able to provide robust communication links. Based on experimental results collected from a testbed, Rahman and Suryanegara discussed the channel orthogonality for different spreading factor values [22]. Radio interference in the 868 MHz band was also studied [24], being shown that communication coverage drops exponentially as the number of end-devices grows.

Other specific aspects related to LoRa performance evaluation concern network scalability. The dimension of a LoRa network was discussed by Mikhaylov et al. and by Bor et al. [25,26]. These authors provide an analysis of the throughput available to a single node and of the number of devices which can be served by a single gateway.

Concerning LoRa energy consumption, there are several works addressing this issue [27,28,29]. These aim at modeling the energy performance of LoRa to characterize the lifetime of specific setups. In this paper, we provide a different model for estimating node energy consumption for LoRa communication technology. This model also includes the computation time, that is not included in previous models. Moreover, we apply and validate it using data from a real setup.

In terms of performance evaluation of LoRa for different types of application scenarios in real deployments, Persia et al. evaluate LoRa for a rural smart grid application [30], looking at network coverage and path losses. According to the authors, the packet delivery success ratio ranges from 90% to 95%. Haghi et al. report a study on tracking human movements (walking movements) within a certain area covered by LoRa [31]. According to them, it is not feasible to track a person on the streets in an Urban area. The authors claim that 200 m is the successful packet reception range instead of 25 km reported by theoretical models. The work by Petri$\overset{´}{c}$ et al. evaluates the performance of LoRa in an urban environment [32], reporting an uncertain and variable network behavior, with packet error rates varying from 3% to 90% without any configuration changes. LoRa performance results in real environments [33] have also been reported. The authors have shown that LoRa enables communication to distances larger than 10 Km, but only if considering transmissions in line-of-sight. Authors also conclude that the maximum transmission range is severely affected by obstructions such as buildings and vegetation. The importance of the spreading factor parameter was quantified, resulting in a recommendation to select a lower spreading factor whenever possible. The same conclusions were also reached by Oliveira et al. and by Petajajarvi et al. [16,17].

Differently from the above-mentioned works, we consider an estuary monitoring scenario where communication is done over water. In particular, and differently from most of the existing works, we consider nodes located at low heights, below 2 m, and we obtain experimental data that allows evaluating communication reliability in this scenario and conditions. Furthermore, we also obtain results on a countryside scenario for comparison purposes. In this sense, our work and results complement the existing knowledge on the application of LoRa, allowing us to conclude that existing empirical propagation models do not adequately represent the reality for such low communication heights.

There is also research related to LoRa transmission over water. The work by Parri et al. discusses the realization of a LoRaWAN network infrastructure for marine environments like an aquaculture industrial plant [34]. signal-to-noise ratio (SNR) and received signal strength indicator (RSSI) were evaluated considering different network parameters and two antenna heights. The reported results achieved a communication link up to 8.33 km with antennas placed always 2.1 m above water level. A similar work was conducted by Jovalekic et al. [35], investigating the behaviour of LoRa in terms of RSSI and SNR, considering three different LoRa spreading factors, within a bandwidth of 125 kHz and 4/6 coding rate. Antennas were placed, at minimum, 56 m above sea level. Their experiments showed that in the conditions considered, LoRa links are fully feasible over seawater at distances of at least 22 km.

Li et al. report the application of LoRa in a sailing monitoring system [36] and Agbuya et al. describe the construction of a tidal monitoring system based on cloud services [37]. In these works the evaluation of network parameters is done taking into account the requirements of the application. In the first case, the transmitter is placed in a boat with the antenna 1.5 m above the water level. The authors report a coverage between 3 and 5 km depending on the spreading factor. In the second case, the most similar to our work, the LoRa transmitter is placed at a small height in a buoy. The results show a small range coverage of about 306 m. Our results show roughly the same behaviour at this height, but in addition we obtain results for a wider range of heights.

As mentioned, we consider that sensor nodes and their respective antennas must be located at low heights, close to the water but in the shore (without buoys or specific platforms like a boat). This represents a different perspective when compared to the work analysed above, in which antennas are deployed at higher positions, thus providing more cleared Fresnel zones [38] and increasing the communication range of LoRa. Concerning the work by Li et al. and by Agbuya et al. [36,37], floating platforms were used. Therefore, the antenna heights do not change with tides, and the variation of communication reliability over time is not investigated, as in this paper.

According to our best knowledge, our work is partially different from the state-of-the-art since it considers over-the-water communication with antennas of nodes placed at low heights (near the water), demonstrating that LoRa performance is affected by factors such as tides and low antenna heights.

## 3. LoRa Characteristics

LoRa is supported by the unlicensed radio bands, promises kilometers of communication distance and several years of battery life. It uses the Chirp Spread Spectrum technology [18] that, according to Semtech [10], makes it robust against a high degree of interference, multi-path and Doppler effects [39,40].

Lora employs a variation of the Chirp Spread Spectrum modulation, where channel, bandwidth, spreading factor and transmission power characteristics are taken into account. It uses a mechanism for constantly increasing or decreasing frequency that sweeps through and wraps around a predefined bandwidth (upchirps and downchirps) to formulate a full packet.

### 3.1. Spreading Factor

The spreading factor (SF) is consistent throughout the packet and can assume different values (typically, it can assume a value from SF5 to SF12). SF5 is used for high data rates, requiring highest SNR for successful demodulation while SF12 supports lowest data rates with lowest SNR for the same transmission power. Combined with the frequency bandwidth, the speeding factor determines the final data rate.

### 3.2. Bandwidth

Another characteristic of LoRa is the Bandwidth (BW), which determines the width of the transmitted signal and, consequently, the chirp duration.

The relation between the spreading factor and the bandwidth is explained by Semtech [11]. Each chirp or symbol consists of 2^SF^ Radio Frequency (RF) chips carrying SF data bits. The number of chips that compose a complete chirp is directly proportional to the bandwidth. Considering a bandwidth of 125 KHz, a chirp comprises 125,000 chips/s and its duration $T_{sym}$ is given by Equation (1).

(1) $$T_{sym}=\frac{2^{SF}}{BW}.$$

Current LoRa chipsets support bandwidth values of 125 kHz, 250 kHz and 500 kHz with a fixed SF and Gaussian Frequency Shift Keying (GFSK) modulation. By changing the bandwidth, chip duration would change accordingly, affecting the chirp duration and the SNR.

### 3.3. Coding Rate

LoRa utilizes Hamming Code as an error detection and correction mechanism. It is represented as 4/x, which indicates 4 information bits together with x (1 to 4) parity bits. The settings embedded into the LoRa chips are 4/5, 4/6, 4/7 and 4/8. Using a Hamming error detection and correction code induces overhead on the transmission by increasing the number of bits to be transmitted. However, it allows a receiver to check for errors and possibly correct them by exploiting forward error correction (FEC) techniques [41], improving communication reliability.

### 3.4. Packet Structure

**Figure 1:**
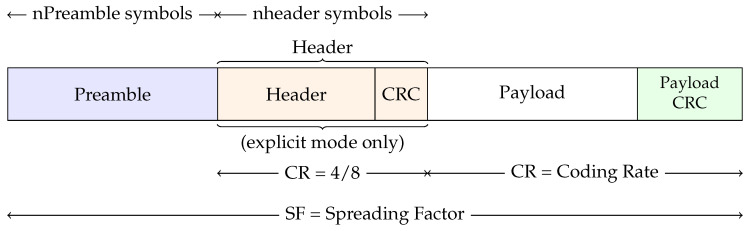
Longe Range (LoRa) uplink packet structure.

There are two types of LoRa packets, each having its own structure—uplink and downlink packets [10]. The difference resides on the presence or absence of a payload cyclic redundancy check (CRC). An uplink LoRa packet (Figure 1) consists of a set of preamble symbols ($Preamble_{Size}$), an optional header, a variable-length payload field and an optional CRC field.

Considering an uplink LoRa packet, the preamble is used to synchronize the receiver with the incoming data. It starts with a sequence of constant upchirps with the last two upchirps encoding the sync word. The sync word is a byte value that is used to identify LoRa networks. A device configured with a specific sync word will stop listening for a transmission if the decoded sync word does not match its configuration. The sync word is followed by 2.25 symbols corresponding to the necessary downchirps. By default, the packet is configured with 8 upchirps plus 2 upchirps and 2.25 downchirps [42]. However, this may be changed by setting a specific register and can vary between 10.25 and 65,539.25.

Concerning the LoRa header, it includes a set of parameters such as Coding Rate (CR), implicit header (IH), low data rate optimization enabled (DE) and payload length. The existence of a payload CRC field at the end of the packet can be controlled through the CRC flag that is also part of the LoRa header. Each header has also its own CRC (4/8) to allow the receiver to discard invalid headers.

In scenarios where the payload, coding rate and payload CRC are fixed or known in advance, the implicit header mode can be invoked. It allows us to reduce the transmission data. In this case, those parameters must be manually configured on both transmitter and receiver.

Lastly, the packet payload is a field with variable-length that contains the data coded according to the specified Coding Rate. LoRa packet size is limited to 255 Bytes.

Each arrow in Figure 1 represents the size of a part of the packet. The size of the overall packet (the number of transmitted symbols) depends on the SF used. The header size and the payload size depend on the amount of information that is sent and on the CR used. For instance, if we choose *n* header symbols as the initial size for the header, the effective number of used symbols will end up increasing according to the CR, which is 4/8 for the header. Concerning the payload, the CR is configurable and may be different from the header CR.

### 3.5. Transmission Power

By increasing transmission power, the signal will have higher chances to reach the receiver even in noisy environments. However, it directly affects the battery lifetime of a LoRa transmitter. Semtech claims that its chipset SX1276 [11] has a sensitivity of −148 dBm and a transmission power output up to +20 dBm. Based on its modulation schemes, it is able to achieve long transmission distances, to show a high degree of interference immunity and to be optimized in terms of energy consumption. However, the maximum transmission power output should respect regional regulations in order to ensure a fair use of the Industrial, Scientific and Medical (ISM) band. Taking into account the operating frequency in Europe (868 MHz), the maximum power output for devices is +14 dBm.

## 4. Analysis of Wireless Propagation Model for LoRa Technology

Currently, there are some empirical propagation models used to estimate packet loss versus distance in urban and sub-urban areas [14,15,43]. However, although the existing models were derived from experimental data using different configurations and propagation environments, there is no model which considers transmission over water surfaces where antennas are placed at low heights in relation to the water surface. Therefore, in this Section we analyse the existing models that will be used to estimate the maximum communication distances achievable with LoRa at different antenna heights.

We begin by presenting an equation that relates the path loss attenuation with the received power. After that, and by setting a minimum value for the received power that still allows communication, it will be possible to establish a relation between the height at which sender and receiver antennas are placed and the communication distance. Taking into account the transmission power ($T_{Pow(dBm)}$), the antenna gains for transmitter and receiver ($G_{Tx}$ and $G_{Rx}$, respectively), and the path loss attenuation ($L_{Atn}$) caused by the distance, the received power ($R_{Pow(dBm)}$) can be calculated as:

(2) $$R_{Pow(dBm)}=T_{Pow}+G_{Tx}+G_{Rx}-L_{Atn}.$$

There are several models to estimate $L_{Atn}$ (e.g., References [12,44,45,46]). However, some of them are simplified models and none was designed to consider water environments associated with low antenna height. For example, the Erceg model [44] tends to overestimate the distances in an urban environment because buildings and other urban structures are not considered by the model [47]. Lee propagation model [45], adapted and evaluated by Dobrilovi$\overset{´}{c}$ et al. [46], can also be used to predict the path loss for point-to-point communications over flat terrain. The application of the Lee propagation model to water environments requires a specific set of parameters that are not trivial to calculate in these environments. The empirical Okumura-Hata model [12] is another option to estimate $L_{Atn}$. This model was developed for wireless communications in urban environments and takes into account the frequency, the transmitter and receiver antenna heights, a correction parameter that depends on the surrounding environment, and the distance between transmitter and receiver.

An alternative to estimate $L_{Atn}$ is the two-ray ground reflected model [13]. It is a radio propagation model which predicts the path losses between a transmitting antenna and a receiving antenna when they are in line-of-sight and each have different height. The model considers received signals having two components, the line-of-sight component and the multi-path component formed by a single ground reflected wave. Since in this experimental work communications in line-of-sight are always considered, we will estimate the path loss in water environments considering the Okumura-Hata model and the two-ray ground reflected model.

The Okumura-Hata model has two parameters that modulate the environment (urban, sub-urban or rural). In our case, since the bay in which we performed the experiments is surrounded by buildings and other infrastructures, we configured the model for sub-urban environments. In terms of terrain profile, the Okumura-Hata model does not include any factor to modulate it. However, given that all our experiments were performed on flat terrain in line-of-sight, the model can be considered appropriate as a reference. In fact, this was confirmed by our results, since the estimations (values resulting from the model) are close to the values obtained in our measurements. The path loss Equation for the Okumura-Hata model is written as [48]:

(3) $$L_{Atn}=69.55+26.16*log_{10}\left(F_{c}\right)-13.82*log_{10}\left(h_{GW}\right)-a\left(h_{node}\right)+\left(44.9-6.55*log_{10}\left(h_{GW}\right)\right)*log_{10}d,$$

where $F_{c}$ is the carrier frequency, which varies from 150 MHz to 1500 MHz, $h_{GW}$ is the gateway antenna height, and *d* is the distance between transmitter and receiver, in kilometers.

$a(h_{node})$ is a parameter that modulates the influence of the environment and the height of node ($h_{node}$) in the communication. Since transmitter and receiver nodes may have different heights, the distance between them is different from that of a single line-of-sight with nodes at the same height. It is given by Equation (4).

(4) $$a\left(h_{node}\right)=3.2*{\left[log_{10}\left(11.75*h_{node}\right)\right]}^{2}-4.97.$$

This model does not consider the terrain profile and the propagation over water or land is not distinguished. However, we considered this model as a good starting point to estimate the maximum communication distance between two LoRa nodes.

Other possibility to determine the path loss is considering the two-ray model which takes into account the fact that radio propagation signals will suffer attenuation (constructive and destructive) due to its own ground reflection. Path loss is often estimated assuming free space propagation, taking into account only the distance *d* and the wavelength λ:

(5) $$L_{Atn}=20\log_{10}\left(4\pi \frac{d}{\lambda }\right).$$

**Figure 2:**
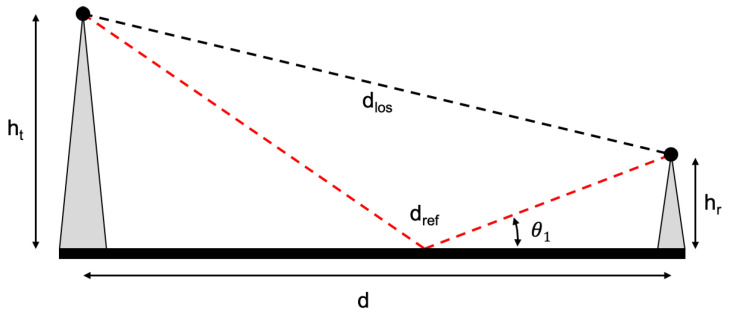
Representation of ground reflection causing distance-dependent constructive and destructive signal interference considered by the two-ray model.

Considering the scenario presented in Figure 2, the length of the direct line-of-sight propagation path can be geometrically derived to be,

(6) $$d_{los}=\sqrt{d^{2}+{\left(h_{t}-h_{r}\right)}^{2}}\;,$$

while the length of the indirect, non line-of-sight path via ground reflection can be described as:

(7) $$d_{ref}=\sqrt{d^{2}+{\left(h_{t}+h_{r}\right)}^{2}}.$$

Based on the length difference of these paths and the wavelength, the phase difference of interfering rays can be derived as:

(8) $$\varphi =2\pi \left(\frac{d_{los}-d_{ref}}{\lambda }\right).$$

The attenuation of radio signal due to reflection is commonly captured in a reflection coefficient, which is dependent on the incidence angle $\theta_{i}$. In order to determine the reflection coefficient, the sine and cosine of $\theta_{i}$ need to be known. They are determined as:

(9) $$sin\theta_{1}=\frac{\left(h_{t}+h_{r}\right)}{d_{ref}}$$

(10) $$cos\theta_{1}=\frac{d}{d_{ref}}.$$

Finally, the reflection coefficient can be calculated as,

(11) $$\Gamma =\frac{sin\theta_{1}-\sqrt{\epsilon_{r}-cos^{2}\theta_{1}}}{sin\theta_{1}+\sqrt{\epsilon_{r}-cos^{2}\theta_{1}}}\;,$$

where $\epsilon_{r}$ is a constant that represents the relative permittivity of the of reflection surface. The modification in signal strength due to constructive or destructive interference can then be modeled by:

(12) $$L_{Atn}=20\log_{10}\left(4\pi \frac{d}{\lambda }{\left|1+\Gamma e^{\varphi }\right|}^{-1}\right).$$

Equation (12) is based on the free space propagation model, taking into account the distance *d* and the wavelength λ, where a correction term of the relative phase and magnitude of interference by the reflected ray was added [49]. This two-ray model describes the received signal strength as the interference of only two copies of the transmitted signal, one that follows the line-of-sight ray and a second that is reflected on the reflection surface (ground or water).

In the next sub-Sections we will estimate the maximum distances that can be achieved for specific heights of the gateway and the node antennas, considering Okumura-Hata and two-ray models. A transmission power of 14 dBm, an operating frequency of 868 MHz and antenna gains of 1.5 dBi and 1 dBi for gateway and nodes, respectively, were considered.

### 4.1. Okumura-Hata Model

**Figure 3:**
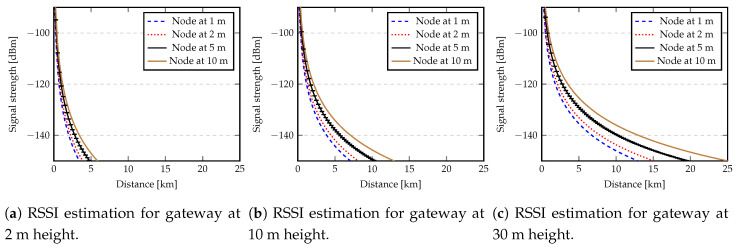
Evolution of $R_{Pow}$ taking into account different distances between gateway and node.

Figure 3 shows the evolution of $R_{Pow}$ for a varying distance between the gateway and a node. The gateway was fixed at 2, 10 or 30 m height, while the node height was set to 1, 2, 5 and 10 m.

From the results shown in Figure 3 we can conclude that if a gateway is fixed at 30 m and a node is set to 10 m, then the maximum communication distance would be up to 25 km, which matches results presented in the literature (e.g., References [16,17,33]). However, the maximum distance is highly reduced for low heights.

Given the focus of this work in studying the reliability of LoRa when nodes are positioned at low heights (0.3, 1 and 2 m), the gateway is fixed at 4 m and the communication is done over the water surface, we used this model to estimate the maximum communication distances. Although the sensitivity of a LoRa receiver can be up to −148 dBm [11], since retransmission and FEC mechanisms were not used in our experiments, we noticed that messages arriving at gateway or nodes with a sensitivity less than −130 dBm, had errors and thus were discarded. Due to this, a reasonable value for $R_{Pow}\left(dBm\right)$ was defined as −130 dBm. Table 1 provides the obtained values for the maximum communication distance *d* in relation to the node height.

To determine the values in Table 1 we used the same transmission power, frequency and antenna gains used to obtain the values depicted in Figure 3. These values were the same that we used in our setup for real measurements.

### 4.2. Two-Ray Model

One important measure that is considered in the two-ray model is the cross-over distance $d_{c}$, that is, the distance where the second ray is reflected. This distance can be derived as:

(13) $$d_{c}=4\pi \frac{h_{t}h_{r}}{\lambda }.$$

If the distance between the gateway and a node (*d*) was less than $d_{c}$, only the direct ray is considered and $L_{Atn}$ is determined by Equation (5), otherwise Equation (12) is applied. Figure 3 shows the evolution of $d_{c}$ for node heights 1, 2, 5 and 10 m, while the gateway was fixed at 2, 10 or 30 m height.

**Figure 4:**
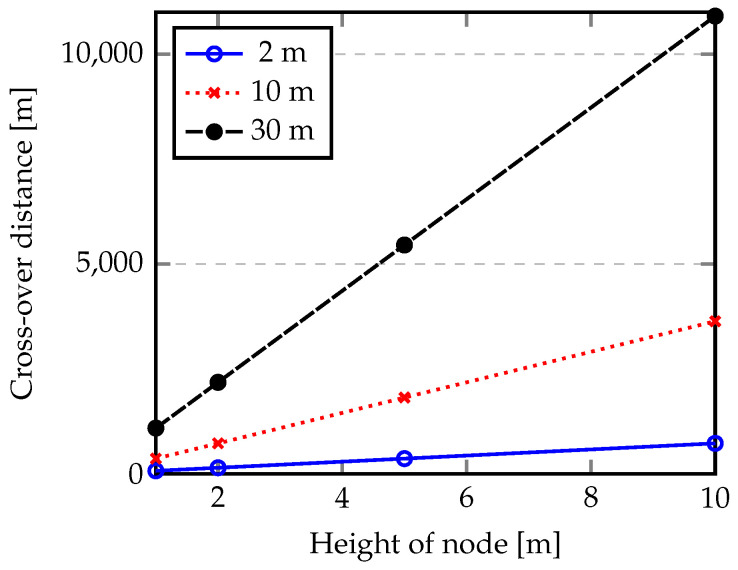
Evaluation of cross-over distance according to heights of the gateway and nodes.

From the results shown in Figure 4 we can conclude that if a gateway is fixed at 30 m and a node is set to 10 m, then the cross-over distance is 10,907 m. On the other hand, if low heights were considered for the gateway and nodes (e.g., gateway at 2 m and node at 1 m), the cross-over distance occurs at 72.72 m.

As discussed before, the focus of this work is studying the reliability of LoRa when nodes are positioned at low heights (0.3, 1 and 2 m) and the gateway is fixed at least 4 m above the water surface. Table 2 provides the results for the cross-over distance in relation to the node heights.

From the results shown in Table 2 we can conclude that, taking into consideration the characteristics of the setup, the cross-over distance is small for long-range communications and Equation (12) must be used to estimate $L_{Atn}$ and, consequently, the maximum distance between the gateway and a node.

Given the same conditions used in Okumura-Hata model (nodes are positioned at low heights—0.3, 1 and 2 m—the gateway is fixed at 4 m and the sensitivity value less than −130 dBm), Table 3 provides the obtained values for the maximum communication distance *d* in relation to the node height when the two-ray model is applied (Equation (12)).

## 5. Experimental Setup

**Figure 5:**
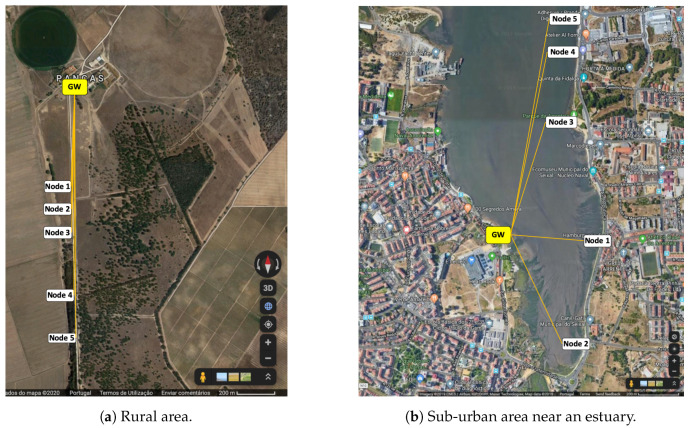
Physical location of nodes.

In order to evaluate the reliability of LoRa in an estuary flooding scenario we built two experimental setups. The first was built in a rural area, far from the estuary to avoid water influences, to collect data (serving as a kind of ground-truth) relative to transmission quality over solid land. The second scenario, the target one, was built in a sub-urban area in the Tagus river estuary, requiring communication over the water and subject to varying water levels. Data collected from the two setups was used to evaluate—(a) the quality of LoRa communication when considering small node heights in relation to the water and the ground and; (b) the influence of tide on the communication quality. This evaluation was motivated by the dependability requirements imposed by the AQUAMON project, a research project that aims to develop a platform for dependable monitoring in water environments [50]. Figure 5 shows the locations where nodes were placed in both scenarios.

Each network comprises five nodes and a gateway. The gateway to node distances are presented in Table 4.

In all experiments, the position of the gateway is fixed at 6.8 m above the ground. In the case of the estuary scenario (Figure 5b), this height varies from 4 to 6.8 m, depending on the tide. The heights of nodes were set at 30 cm, 1 m and 2 m above the ground level for the rural scenario (Figure 5a) and above the water for the estuary scenario (Figure 5b). In the estuary scenario, node positions were continually adjusted over time to ensure that despite water level changes each new data acquisition task (lasting for 15 min) would be done with the nodes at the desired height.

In terms of network evaluation, measurements of signal strength, signal-noise ratio, packet loss and round-trip time were collected. A simple request-reply communication protocol between the gateway and the nodes was used. The gateway acts as a master, sending a request packet to each node and receiving the corresponding reply. Given the scenarios, the experiments were done separately. In both, the defined communication protocol ensures that no packet collisions occur because just one request is sent at a time. A timeout of 2 seconds is used to wait for the reply. When a timeout occurs, the message reply is considered as lost. All received replies were stored in the gateway to be processed later on.

In terms of LoRa packet configuration, only uplink packets were used with an explicit header and payload CRC verification. Concerning the payload, it was different for request and reply packets. Each request packet had a payload of 20 bytes, while a reply packet was composed of 85 bytes, where performance data and node coordinates, given by a GPS receiver, were included.

Concerning the LoRa chipset configuration, the maximum allowed transmission power over 868 MHz was used (+14 dBm), with an SF12 spreading factor, a coding rate of 4/5 and a bandwidth of 500 kHz.

According to EU regulations, the allowed bandwidth for frequencies in the range of 25 MHz ≤f≤ 1000 MHz is 100 kHz or 120 kHz [51]. However, given that the LoRa technology supports a higher bandwidth of 500 kHz (leading to improved performance), we decided to run the experiments using this 500 KHz bandwidth for the purpose of evaluating the best case, not a restricted and particular one.

In terms of antenna gains, the gateway antenna has 1.5 dBi while nodes have a unitary gain antenna (1 dBi).

Many experiments were conducted over the course of a few months. Each experiment consisted in:

1. placing the gateway and nodes in specific locations at the desired initial height;
2. executing the described protocol during 15 min (in the estuary case, during the high tidal level we collected measurements during 20 min in order to increase the number of samples. In general, no statistical difference was observed on the packet loss.);
3. placing the nodes at a new height above the ground and repeating the process from (2).

**Figure 6:**
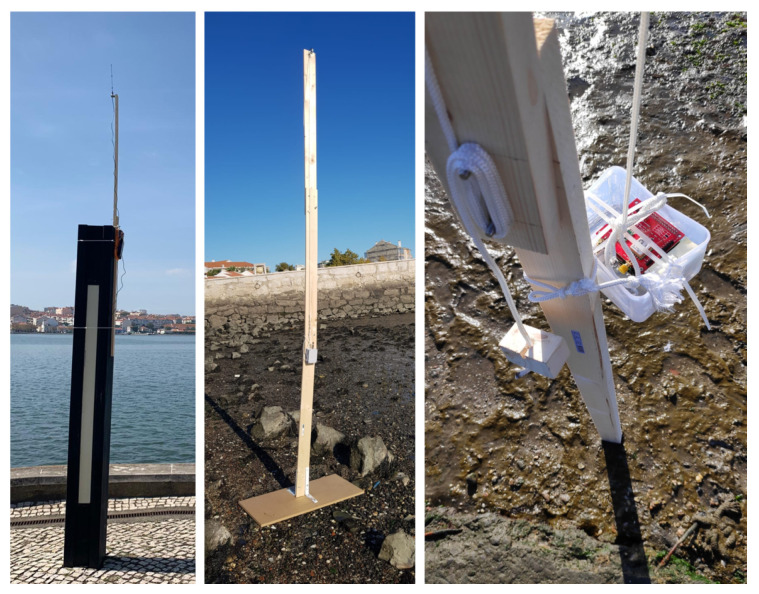
Gateway and sensor nodes in the monitoring field.

Figure 6 shows, on the left, the gateway and, on the middle and right, Arduino and Raspberry PI nodes mounted on a pole with height marks. The gateway is also a Raspberry PI that executes the master role of the described protocol.

Since we are interested in flood prevention, the variation of tidal level needs to be taken into account. In our scenario, the average amplitude of the tide varies from 0 m in low tide to 2.8 m in high tide. In the context of this work, when we refer to low or high tide we consider a time span of about 2 h around the lowest and highest water levels, respectively.

## 6. Analysis of the First Fresnel Zone Clearance

Radio frequency waves propagate not only directly along the line of sight path, but also in an off-axis fashion. Reflections caused by obstacles to the propagation will cause phase variations on the reflected signals. According to Parri et al. [34], if direct and reflected signals take opposite phases (i.e., there is a change of phase to (2k+1)π with k∈N in the reflected signal), signals will be cancelled out at the receiver side, originating losses. If signals are perfectly in phase (there is a change of phase equal to 2kπ with k∈N), the signal will be enhanced at the receiver side. Naturally, intermediate situations can also occur. In general, reflections should be avoided since phase changing ordinarily leads to destructive interference and signal weakening. According to He et al. and Green and Obaidat [52,53], line of sight between transmitter and receiver is not theoretically sufficient to assure adequate wireless transmissions. Ideally, any obstruction within the first Fresnel zone should be avoided but this is unfeasible in most real world scenarios. As the phase shifts caused by obstructions in the first Fresnel zone are not large, these can be tolerated as long as at least a 60% clearance is maintained [52,53].

**Figure 7:**
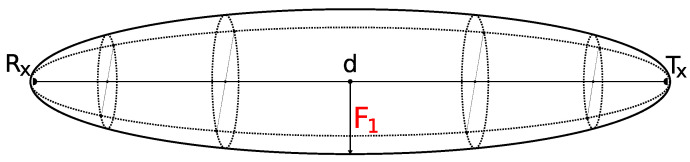
Representation of the first Fresnel Zone.

Figure 7 provides an illustration of the first Fresnel zone: *d* is the distance between the transmitter and the receiver (line of sight) while $F_{1}$ is the radius of the zone, whose maximum occurs at d/2.

If *d* is specified in km and the transmission frequency *f* is defined in GHz, $F_{1}$ is calculated as:

(14) $$F_{1}=8.656\sqrt{\frac{d}{f}}\;.$$

Since the first Fresnel zone can be obstructed by the Earth curvature, the maximum Earth bulge height (*H*), which is experienced in the midpoint of the link, is computed as,

(15) $$H=\frac{375d^{2}}{4R}\;,$$

where *d* is the distance between the transmitter and the receiver in km, and *R* is the Earth radius. For long transmission distances the Earth curvature bulge influences the value of *d*. However, given that in our work the distance is under 1.5 Km, this influence can be neglected (and hence *d*, in Equation (15), represents the distance in line-of-sight without taking into account the Earth curvature influence).

Given that the transmitting and receiving antennas were installed at different heights, the percentage of clearance of the first Fresnel zone is calculated as,

(16) $$F_{1_{C}}=\frac{100\left[\left(\frac{h_{R_{x}}-h_{T_{x}}}{2}\right)-H\right]}{F_{1}}\;,$$

where $h_{R_{x}}$ and $h_{T_{x}}$ represent the heights of transmitting and receiving antennas, respectively. Taking into account the setup described in Section 5, where distances between nodes and gateway are represented in Table 4, nodes are placed at 30 cm, 1 m and 2 m, and the gateway antenna height may vary from 4 to 6.8 m, the first Fresnel zone radius ($F_{1}$) determined by Equation (14) is represented in Table 5.

**Figure 8:**
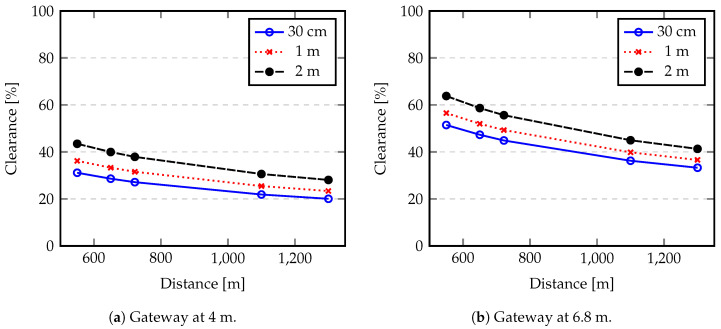
Percentage of clearance of the first Fresnel zone according to the gateway antenna height.

In our setup, the first Fresnel zone radius varies from 6.89 to 10.59 m. However, this value is reduced by any obstruction within the first Fresnel zone. Figure 8 shows the percentage of clearance of the first Fresnel zone according to the nodes and Gateway antenna heights.

From Figure 8a we can see that the most favourable clearance happens when a node is placed at 2 m (maximum considered height) and closer to the gateway (550 m). However, the result is about 43%, which is still below the recommended value of 60% of clearance to achieve a high communication reliability degree. However, this 43% of clearance may be enough to right decode the transmitted information if it was received with a good SNR. The clearance value increases when the gateway antenna is placed at 6.8 m height (Figure 8b). In this case, 63% of clearance is achieved for maximum node antenna height and minimum communication distance. From this analysis we can conclude that the scenario conditions are not ideal to achieve a high degree of reliability. Additionally, these conditions will be degraded when floods occur since the free space bellow nodes and gateway antennas will decrease, introducing further obstruction within the first Fresnel zone.

## 7. Results and Discussion

In this Section we present and discuss the results obtained from our experiments. The objective is to obtain reliability indicators, such as received packet ratio, received signal strength, signal to noise ratio and round-trip time, for several node-gateway distances and node heights. These indicators were collected for:

- a scenario where a flat terrain is considered without the presence of water (Figure 5a);
- a scenario where the communication is done over the water surface (Figure 5b).

In the water scenario we evaluated the reliability indicators for both high and low tide conditions.

**Figure 9:**
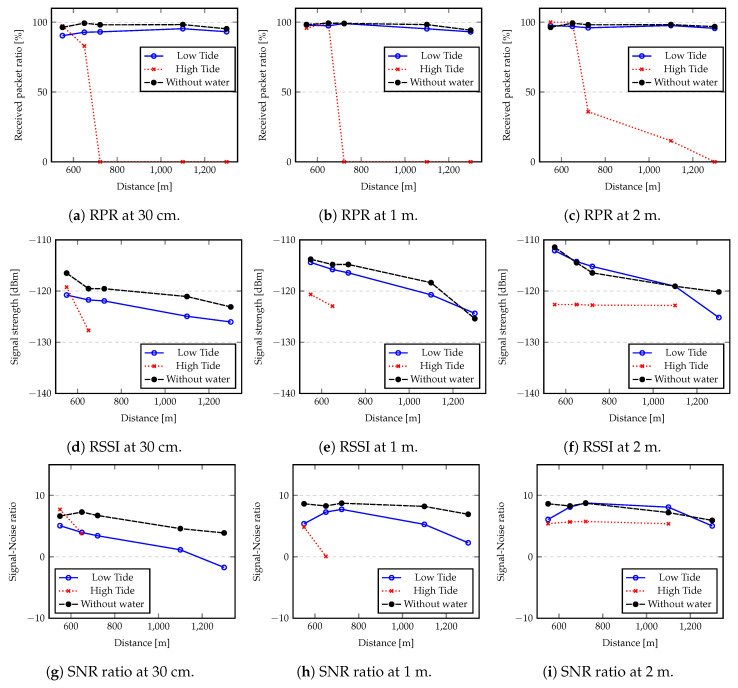
Received packet ratio (RPR), received signal strength indicator (RSSI) and signal-to-noise ratio (SNR) with nodes placed at 30 cm, 1 m and 2 m above water and above the ground (without water).

Figure 9a–c shows the received packet ratios considering the flat terrain and the estuary in low tide and high tide conditions, for antennas at 30 cm, 1 m, and 2 m heights, respectively. For flat terrain and low tide conditions, the results show that the maximum communication distances are close to the ones estimated by the Okumura-Hata model (see Table 1).

However, in high tide conditions, the maximum communication distance drops significantly, reaching only about 650 m for nodes at 30 cm or 1 m height. Considering the flat terrain and the estuary in low tide conditions (which is similar to flat terrain), 98% of packets were successfully delivered (117 out of 120 packets were received correctly at the gateway). In high tide, the gateway height decreases in relation to water level and this rate drops significantly.

In fact, in low tide conditions, significant parts of the estuary are almost dry, thus approximating the propagation conditions over flat terrain. These results suggest that tides have a high impact on the communication reliability, since the free space between node antennas and the water surface is varying over time.

In these experiments we also collected data for the RSSI and the SNR, which are shown in Figure 9d–i, for the various antenna heights. The results show that both the RSSI and the SNR in low tide are closer to those obtained in flat terrain, in accordance with the measured received packet ratios. As expected, the results obtained with 2 m height antennas are always better than results from lower height antennas.

**Figure 10:**
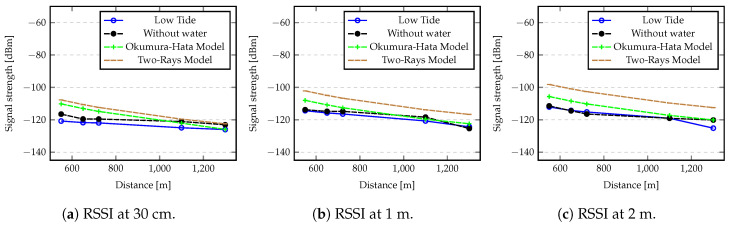
Comparison between experimental and theoretical RSSI values with nodes placed at 30 cm, 1 m and 2 m for ground and low tide conditions.

Figure 10 shows the comparison between the RSSI values determined by Equation (2), considering the Okumura-Hata model and the two-ray model, and the values gathered from the experiments. In all cases all lines follow the same trend, but there is a higher difference for short distances, as the values resulting from the theoretical model are higher for the shorter distances. This difference may be due to hardware saturation, in terms of radio sensitivity, that reduce the received signal strength. However, the difference is mitigated when RSSI values are below to −116 dBm. In this case we can say that the values collected from our experiments are in accordance with the theoretical model, demonstrating the capabilities of the technology to ensure high degree of reliability.

Two main conclusions can be taken from these results—the antennas height influences all the performance indicators; the tide level has a significant impact on the communication reliability when considering antennas placed at low heights.

Regarding the flood alert system scenario, from our experiments we conclude that to deploy such system close to the water, the tidal level must be taken into consideration to keep a minimum height (in our case, 2 m, for a gateway positioned at least 4 m above the water) between node antennas and water surface, sufficient to achieve a desired communication range.

## 8. Battery Lifetime Estimation of a LoRa Node

To achieve dependable remote environmental monitoring applications, several aspects must be considered. These applications can fail for several reasons and two of the most relevant failure modes concerning the communication infrastructure consist of intermittently lost messages (omissions) and permanent loss of communication (e.g., due to crashed nodes). In the previous Sections we analysed the impact of node positioning with respect to the water level on message loss. In this Section we turn our attention to the analysis of the battery lifetime of a node, given that battery depletion is a primary reason for permanent failures. A careful characterization of battery lifetime is hence fundamental to raise confidence that a node keeps operational for a certain amount of time. We thus present a method for estimating the energy consumption of a LoRa node.

The battery lifetime can be estimated by Equation (17):

(17) $$Node_{Lifetime}=Period*\frac{E_{battery}}{E_{cycle}}.$$

The parameter Period represents the time duration between consecutive wake-ups. Parameters $E_{battery}$ and $E_{cycle}$ refer to the energy contained in the node battery and energy expended for each Period, respectively. The lifetime calculated does not assume battery degradation factors due to time or environmental influences.

The energy contained in a node battery can be obtained through Equation (18), where the value of 3600 corresponds to the number of seconds per hour, $C_{battery}$ is the charge in the battery (Ampere per hour (Ah)), and $V_{battery}$ is the voltage of the battery.

(18) $$E_{battery}=3600*C_{battery}*V_{battery}.$$

Taking into account the packet structure discussed in Section 3, the Time on Air (ToA) ($T_{TX}$) can be calculated using Equation (19), where the time per symbol $T_{sym}$ is given by Equation (1), the number of preamble symbols is defined by the user ($Preamble_{Size}$), and the number of payload symbols ($Payload_{Size}$), according to the Semtech datasheet [11], can be calculated by Equation (20). The $Preamble_{Size}$ must include the 2 mandatory synchronization symbols and 2.25 symbols of Start Frame Delimiter.

(19) $$T_{packet}=T_{sym}*\left(Preamble_{Size}+Payload_{Size}\right)$$

(20) $$Payload_{Size}=8+max\left(\left\lceil \frac{2*PL-SF+2+4*CRC+5*IH}{SF-2*DE}\right\rceil *\left(CR+4\right)\right).$$

In Equation (20), PL represents the payload in bytes, SF represents the spreading factor, CR represents the coding rate, IH indicates explicit (1) or implicit (0) header, and both CRC and DE indicate absence (0) or presence (1) in the packet.

Since a node sends data every Period and the remaining time is used for computations, receive data and sleep, $E_{cycle}$ can be divided into four parts, the energy expended during idle state ($E_{cycle_{idle}}$), the energy expended to do in-node computations ($E_{cycle_{comput}}$), the energy expended during the transmission state ($E_{cycle_{TX}}$) and the energy required to receive a data packet ($E_{cycle_{RX}}$). Equation (21) allows us to obtain the amount of energy expended per Period using LoRa.

(21) $$E_{cycle}=E_{cycle_{idle}}+E_{cycle_{comput}}+E_{cycle_{RX}}+E_{cycle_{TX}}$$

where

(22) $$E_{cycle_{idle}}=\left(Period-\left(T_{packet_{RX}}+T_{packet_{TX}}+T_{Comput}\right)\right)*\left(MCU_{idle}+Radio_{OFF}\right)$$

(23) $$E_{cycle_{Comput}}=T_{Comput}*\left(MCU_{ON}+Radio_{OFF}\right)$$

(24) $$E_{cycle_{RX}}=T_{packet_{RX}}*\left(MCU_{ON}+Radio_{RX}\right)$$

(25) $$E_{cycle_{TX}}=T_{packet_{TX}}*\left(MCU_{ON}+Radio_{TX}\right).$$

With $T_{packet_{RX}}=T_{packet_{TX}}=T_{packet}$ if both reception and transmission were considered in a single cycle. If just a transmission is considered, $T_{packet_{RX}}$ must be zero, while if a single reception is considered without data transmission, $T_{packet_{TX}}$ must be considered zero.

**Figure 11:**
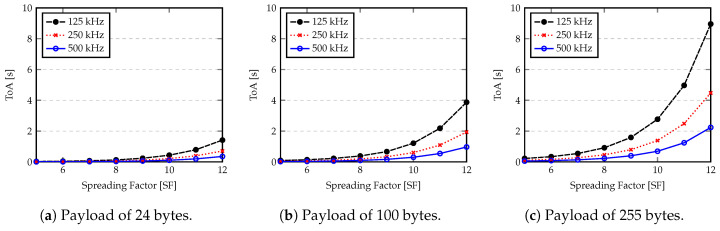
Packet Time on Air (ToA) for different payload length according to Bandwidth and Spreading Factor.

In order to demonstrate the influence of LoRa setting parameters, Figure 11 shows the ToA for different payload length (in bytes), using several combinations of bandwidth and spreading factors. These values are obtained by applying Equation (19).

As expected, payload length plays an important role on the ToA. However the combination of a right bandwidth and spreading factor can optimize that time. Since ToA is directly related to the amount of energy that a node needs to expend to transmit the data packet, it is an important aspect to determine the battery lifetime of a node.

**Figure 12:**
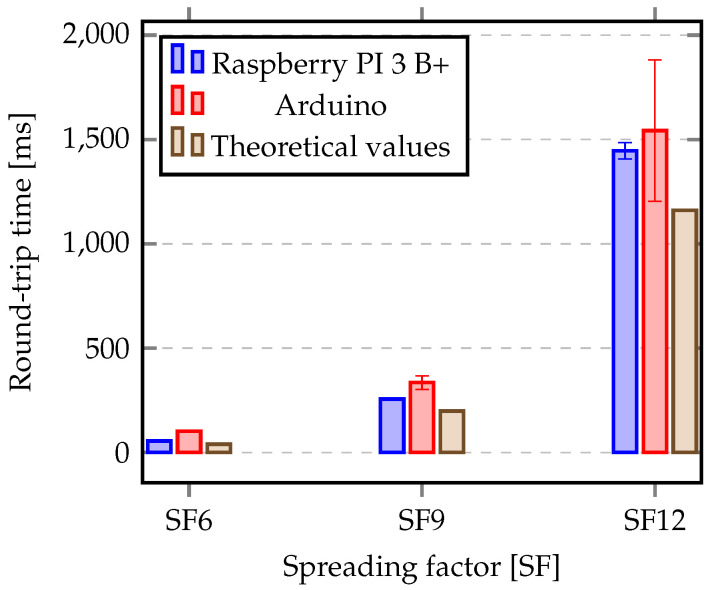
Round-trip time (average and standard deviation) evaluation for different spreading factors.

As described in Section 5, for our measurements a bandwidth of 500 kHz is configured and a request-reply protocol for the communication between the gateway and the nodes is used, where each request has 20 Bytes and the reply has 85 Bytes. All request messages include a time-stamp from the gateway. When a node receives the request it builds a response message where initial time-stamp is included. Upon reception of a reply at the gateway, the round-trip time is computed. Figure 12 shows the average and standard deviation of the round-trip time for 117 successfully delivered packets at the gateway.

Due to the specific characteristics of the second evaluation scenario (a sub-urban area in the Tagus river estuary, requiring communication over the water) a high SF was used to try to improve the communication reliability. However, three different SFs were tested in the rural scenario. In order to compare both scenarios, in terms of signal strength, signal-noise ratio and packet loss, just the SF12 was used. Additionally, since an analysis of LoRa energy consumption was performed in terms of theoretical models, we compared the theoretical values with those obtained from our experimental setup in the rural area. This is the reason why we include three different SF values in Figure 12.

This round-trip time includes the time to deliver the request, the time to process it, the time to build the reply, the time to send the reply and receive it at the gateway.

Table 6 shows the estimation ToA for a request with 20 Bytes and a response with 85 Bytes, considering three SF (SF6, SF9, SF12). As expected, the time needed to transmit a response is higher when compared to the request. Each response takes about three times more than a request. Table 6 also shows the round-trip time, obtained by the sum of ToA for a request and the ToA to receive the response. This round-trip-time is also represented in Figure 12.

In terms of standard deviation, in general, it is small for Raspberry PI node, achieving 1.52 ms for SF6 and SF9, and 40 ms for SF12, representing a stable communication. When an Arduino node is considered, the standard deviation is also small for the lower SF (1.64 ms). However, it is higher for other SF values, achieving 324 ms for SF12. This demonstrates that our setup is working as expected and we can estimate the battery lifetime of nodes according to the theoretical model.

In order to determine the amount of energy expended between each transmission we need to analyse the energy characteristics of the hardware. The characteristics of the hardware used in our setup are represented in Table 7 and Table 8. They were extracted from the hardware data-sheets [11,54,55,56].

**Figure 13:**
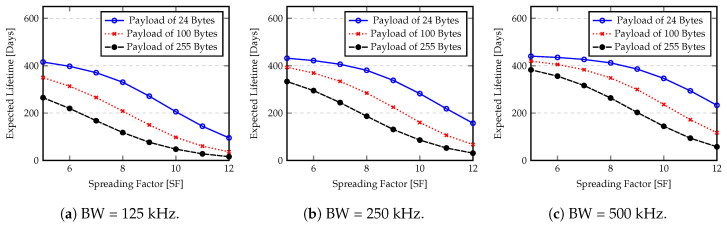
Expected battery lifetime for Arduino node.

**Figure 14:**
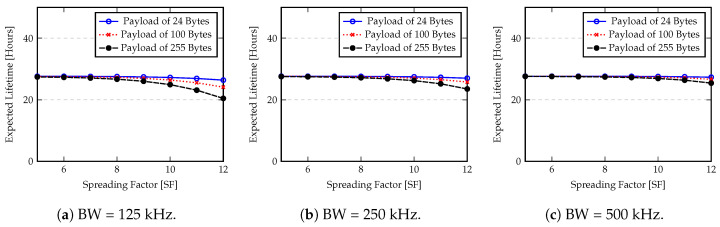
Expected battery lifetime for Raspberry PI 3 B+ node.

Figure 13 and Figure 14 show the expected battery lifetime obtained by Equation (17) for different bandwidth, spreading factor and payloads. For these computations we assume a $Preamble_{Size}$ of 6 symbols plus the mandatory sync word and start frame delimiter. The explicit header with the presence of CRC and DE, and a CR equal to 4 are also included. In terms of data sending rate, we assume that each sample is sent out every 60 s with the maximum transmission power (+14 dBm). The capacity of batteries are 4000 mAh and 1200 mAh for Raspberry PI and Arduino, respectively. A self-discharge rate of 2.5% per month is considered.

As expected, MCU power consumption is the major factor that determines the battery lifetime of nodes. The idle state greatly reduces the energy consumption. However, the Raspberry PI shows a reduced battery lifetime because the power consumption of the single board computer (SBC) is high when compared with Arduino. In this case, the radio power consumption is significantly smaller when compared to Raspberry PI consumption in any state (“idle” or “on”) and the advantage of LPWANs is not reflected in this lifetime estimation, resulting in a reduced node battery lifetime. Another important factor is the quantity of information (Payload size) that must be sent and the time duration of the transmission (spreading factor). From Figure 13 and Figure 14 we can see that the spreading factor plays an important role in the battery lifetime. If the spreading factor is increased, for example, from 6 to 12, the node lifetime is reduced to less than half of initial value. In terms of transmission power, the values of Figure 13 and Figure 14 assume the worse case (maximum expended budget per transmission). If a smaller transmission power is considered, the node battery lifetime will be increased.

**Figure 15:**
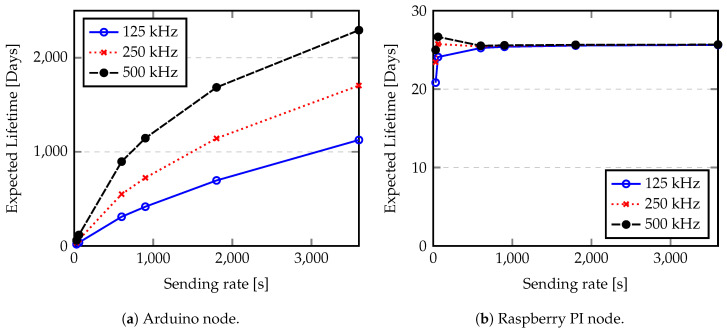
Expected lifetime according to the sending rate.

Since the energy consumption depends on the hardware characteristics, on the time required to do computations, on the quantity of information to be sent, and on the number of transmissions, the node lifetime can be adjusted by applying several energy saving policies. In Figure 13, we show the expected lifetime for an Arduino node taking into account the SF and the payload size. However, if some data fusion techniques [57,58] were applied over data, the node lifetime can be extended. Assuming that data fusion allows us to reduce the sending rate of a node, reducing the energy spent per cycle, the lifetime of a node will be increased. Figure 15 shows the evaluation of expected LoRa node lifetime according to the sending rate for SF = 12 and payload of 100 Bytes. Sending rates of 30, 60, 600, 900, 1800 and 3600 s (0.033, 0.017, 0.0017, 0.0011, 0.0005, 0.0003 Hz) are considered in this analysis.

**Figure 16:**
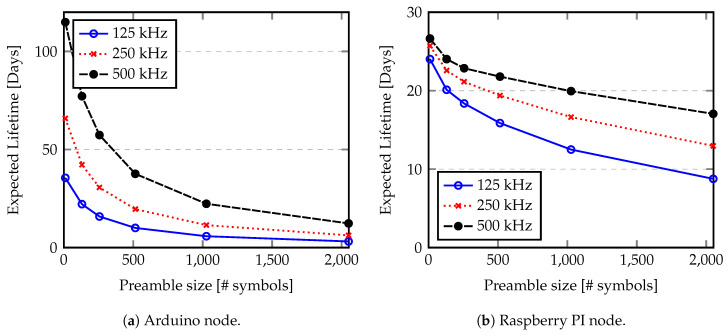
Expected lifetime according to the preamble size.

As we discuss before, the Raspberry PI shows a reduced battery lifetime because the power consumption of the single board computer (SBC) is high. The quantity of energy saved by avoiding data transmissions is reduced and it is consumed quickly by the node itself. However, from Figure 15 we can conclude that sending each message at every 600 s (0.0017 Hz) allows us to optimize the raspberry node lifetime. Since the Arduino node presents a reduced power consumption in idle state and low in processing, the node lifetime in this case can be extended for years. Considering, for example a BW of 125 kHz, we can obtain a variation of the lifetime from 18.2 days for a sending rate of 2 Hz, to 3 years if a sending rate of 0.0003 Hz (1 message per hour) is used. Another characteristic that influences the node lifetime is the length of preamble used by LoRa chips, since it adds some load to the communication. The preamble sampling is used to ensure that a receiver is listening to radio channel and thus able to receive the transmission. Using small preamble makes the communication more susceptible to the external noise, since the receiver can not identify the beginning of the communication and discard the transmission. Alternatively, using large preambles adds more overhead to the communication, thus more energy spent by each transmission. Figure 16 shows the evaluation of expected LoRa node lifetime according to the preamble size. An SF of 12 and a payload of 100 Bytes with one message per minute were used to estimate the node lifetime. Preambles of 10.25 (minimum value), 130.25, 256.25, 514.25, 1026.25 and 2050.25 symbols were considered.

From Figure 16 we can see that the preamble size may have a large impact in the node lifetime since it is considered part of LoRa packet (as defined in Equation (19)).

## 9. Conclusions

In this paper, we presented an analysis of LoRa propagation characteristics in real environments (over land and over water) under different antenna heights, node distances and, in the case of the estuary, considering different tidal water levels. An extensive set of measurements were obtained in the Tagus estuary, near Lisbon, Portugal, and in a countryside scenario with flat and solid terrain, also near Lisbon. The performance of LoRa was evaluated in terms of received packet ratio, RSSI, SNR and network latency.

The reported results support the conclusion that the reliability of LoRa communications in a real environment is dependent on the height at which nodes are placed, and hence varies with tidal levels. When low tidal levels are considered, the achieved communication distances are roughly similar to the forecasts provided by theoretical propagation models, with 117 out of 120 packets successfully delivered, that is, 98% of packets were correctly delivered to the gateway. However, when the water level raises and node heights in relation to the water become very low, theoretical models are no longer adequate and reliability strongly degrades. It is hence possible to conclude that nodes must be carefully positioned, and our results provide empirical data that allows the determination of the lowest heights at which they can still communicate reliably.

We also provide results on LoRa network latency, comparing them to the specifications of a LoRa chip and concluding that the obtained values are according to those specifications. Based on these results, we also derive a battery lifetime estimation model, which allows us to plan the network maintenance.

## Figures and Tables

**Table 1:** Maximum communication distance for different node heights.

Node Height in Relation to Water Surface [m]	Maximum Distance [m]
0.3	1250
1	1450
2	1700

**Table 2:** Two-ray cross-over distance for different node heights.

Node Height in Relation to Water Surface [m]	Cross-Over Distance [m]
0.3	43.63
1	145.43
2	290.87

**Table 3:** Maximum communication distance for different node heights determined by Equation (12).

Node Height in Relation to Water Surface [m]	Maximum Distance [m]
0.3	1350
1	2200
2	3600

**Table 4:** Distances from nodes to the gateway.

Node ID	Distance to the Gateway [m]
1	550
2	650
3	722
4	1100
5	1300

**Table 5:** First Fresnel zone radius.

Distance between Node and the Gateway [m]	First Fresnel Zone (F1) [m]
550	6.89
650	7.49
722	7.89
1100	9.74
1300	10.59

**Table 6:** ToA per message size (request and response) and round-trip time of our experimental protocol.

SF	Payload		Round-Trip Time [ms]
	20 Bytes	85 Bytes	
SF6	10 ms	30 ms	40 ms
SF9	49 ms	150 ms	199 ms
SF12	310 ms	850 ms	1160 ms

**Table 7:** Energy Consumption for different states and MCU used in our setup.

MCU	State	
	Idle	ON
Arduino	3.26 mW	12.40 mW
Raspberry PI 3 B+	1.9 W	5 W

**Table 8:** Energy Consumption for different states of SX1276 radio.

Radio State	Output Budget	Power Consumption
Idle		4.95 μW
Receiving (RX)		39.6 mW
Trnsmmiting (TX)	+20 dBm	396 mW
	+17 dBm	287.1 mW
	+14 dBm	125.7 mW
	+7 dBm	66 mW

## References

[1] Lancellotti E. (2018). Confliting Cases pf Community Resilience to Extreme Weather Events: Evidence from Europe, North America, India and the Philippines.

[2] Kelman I. (2020). Disaster by Choice: How Our Actions Turn Natural Hazards into Catastrophes.

[3] Jongman B., Winsemius H.C., Aerts J.C., de Perez E.C., van Aalst M.K., Kron W., Ward P.J. (2015). Declining vulnerability to river floods and the global benefits of adaptation. Proc. Natl. Acad. Sci. USA.

[4] Cook H.F. (2017). The Protection and Conservation of Water Resources.

[5] Baig M.S.S., Rajalakshmi P. (2019). Wireless Sensor Network for Real Time Pollution Monitoring and Smart Grid Applications. Ph.D. Thesis.

[6] Falih-Al-Khalidi A.M., Al-Asady R.K.A. (2019). Environmental Monitoring of the Shatt Al-Diwaniyah River Water Quality using GSM Wireless Remote Sensing Technology (WSN).

[7] Grover K., Kahali D., Verma S., Subramanian B. (2020). WSN-Based System for Forest Fire Detection and Mitigation. Emerging Technologies for Agriculture and Environment.

[8] Sanchez-Iborra R., Sanchez-Gomez J., Ballesta-Viñas J., Cano M.D., Skarmeta A. (2018). Performance evaluation of LoRa considering scenario conditions. Sensors.

[9] Raza U., Kulkarni P., Sooriyabandara M. (2017). Low power wide area networks: An overview. IEEE Commun. Surv. Tutor..

[10] LoRa Alliance^®^. https://lora-alliance.org/.

[11] Semtech Semtech SX1276. https://www.semtech.com/products/wireless-rf/lora-transceivers/sx1276.

[12] Hata M. (1980). Empirical formula for propagation loss in land mobile radio services. IEEE Trans. Veh. Technol..

[13] Sommer C., Joerer S., Dressler F. On the applicability of two-ray path loss models for vehicular network simulation. Proceedings of the 2012 IEEE Vehicular Networking Conference (VNC).

[14] Harinda E., Hosseinzadeh S., Larijani H., Gibson R.M. Comparative Performance Analysis of Empirical Propagation Models for LoRaWAN 868MHz in an Urban Scenario. Proceedings of the 2019 IEEE 5th World Forum on Internet of Things (WF-IoT).

[15] El Chall R., Lahoud S., El Helou M. (2019). LoRaWAN Network: Radio Propagation Models and Performance Evaluation in Various Environments in Lebanon. IEEE Internet Things J..

[16] Petajajarvi J., Mikhaylov K., Roivainen A., Hanninen T., Pettissalo M. On the coverage of LPWANs: Range evaluation and channel attenuation model for LoRa technology. Proceedings of the 2015 14th International Conference on ITS Telecommunications (ITST).

[17] Oliveira R., Guardalben L., Sargento S. Long range communications in urban and rural environments. Proceedings of the 2017 IEEE Symposium on Computers and Communications (ISCC).

[18] Goursaud C., Gorce J.M. (2015). Dedicated networks for IoT: PHY/MAC state of the art and challenges. EAI Endorsed Trans. Internet Things.

[19] Bor M.C., Roedig U., Voigt T., Alonso J.M. Do LoRa low-power wide-area networks scale?. Proceedings of the 19th ACM International Conference on Modeling, Analysis and Simulation of Wireless and Mobile Systems.

[20] Bor M., Roedig U. LoRa transmission parameter selection. Proceedings of the 2017 13th International Conference on Distributed Computing in Sensor Systems (DCOSS).

[21] Augustin A., Yi J., Clausen T., Townsley W. (2016). A study of LoRa: Long range & low power networks for the internet of things. Sensors.

[22] Rahman A., Suryanegara M. The development of IoT LoRa: A performance evaluation on LoS and Non-LoS environment at 915 MHz ISM frequency. Proceedings of the 2017 International Conference on Signals and Systems (ICSigSys).

[23] Petäjäjärvi J., Mikhaylov K., Yasmin R., Hämäläinen M., Iinatti J. (2017). Evaluation of LoRa LPWAN technology for indoor remote health and wellbeing monitoring. Int. J. Wirel. Inf. Netw..

[24] Lauridsen M., Vejlgaard B., Kovacs I.Z., Nguyen H., Mogensen P. Interference measurements in the European 868 MHz ISM band with focus on LoRa and SigFox. Proceedings of the 2017 IEEE Wireless Communications and Networking Conference (WCNC).

[25] Mikhaylov K., Petaejaejaervi J., Haenninen T. Analysis of capacity and scalability of the LoRa low power wide area network technology. Proceedings of the European Wireless 2016—22th European Wireless Conference.

[26] Bor M.C., Vidler J., Roedig U. LoRa for the Internet of Things. Proceedings of the International Conference on Embedded Wireless Systems and Networks (EWSN).

[27] Mahmoud M.S., Mohamad A.A. (2016). A Study of Efficient Power Consumption Wireless Communication Techniques/Modules for Internet of Things (IoT) Applications.

[28] Casals L., Mir B., Vidal R., Gomez C. (2017). Modeling the energy performance of LoRaWAN. Sensors.

[29] Kazdaridis G., Zographopoulos I., Symeonidis P., Skrimponis P., Korakis T., Tassiulas L. In-situ Power Consumption Meter for Sensor Networks supporting Extreme Dynamic Range. Proceedings of the 11th Workshop onWireless Network Testbeds, Experimental Evaluation & CHaracterization.

[30] Persia S., Carciofi C., Faccioli M. NB-IoT and LoRA connectivity analysis for M2M/IoT smart grids applications. Proceedings of the 2017 AEIT International Annual Conference.

[31] Haghi M., Thurow K., Stoll R. (2017). Wearable devices in medical internet of things: Scientific research and commercially available devices. Healthc. Inform. Res..

[32] Petrić T., Goessens M., Nuaymi L., Toutain L., Pelov A. Measurements, performance and analysis of LoRa FABIAN, a real-world implementation of LPWAN. Proceedings of the 2016 IEEE 27th Annual International Symposium on Personal, Indoor, and Mobile Radio Communications (PIMRC).

[33] Liando J.C., Gamage A., Tengourtius A.W., Li M. (2019). Known and unknown facts of LoRa: Experiences from a large-scale measurement study. ACM Trans. Sens. Netw. (TOSN).

[34] Parri L., Parrino S., Peruzzi G., Pozzebon A. (2019). Low Power Wide Area Networks (LPWAN) at Sea: Performance Analysis of Offshore Data Transmission by Means of LoRaWAN Connectivity for Marine Monitoring Applications. Sensors.

[35] Jovalekic N., Drndarevic V., Pietrosemoli E., Darby I., Zennaro M. (2018). Experimental study of LoRa transmission over seawater. Sensors.

[36] Li L., Ren J., Zhu Q. On the application of LoRa LPWAN technology in Sailing Monitoring System. Proceedings of the 2017 13th Annual Conference on Wireless On-demand Network Systems and Services (WONS).

[37] Agbuya F., Apolinario G.F., Ramos D.M., Villanueva J.M., Zafe P., Hernandez J.A., Coquia J. Design of a Real–Time Ocean Data–Logging Drifter Thru CLOUD Technology for Collecting Tidal Parameters. Proceedings of the TENCON 2018-2018 IEEE Region 10 Conference.

[38] Aref M., Sikora A. Free space range measurements with Semtech Lora™ technology. Proceedings of the 2014 2nd International Symposium on Wireless Systems within the Conferences on Intelligent Data Acquisition and Advanced Computing Systems.

[39] Adelantado F., Vilajosana X., Tuset-Peiro P., Martinez B., Melia-Segui J., Watteyne T. (2017). Understanding the limits of LoRaWAN. IEEE Commun. Mag..

[40] Peng Y., Shangguan L., Hu Y., Qian Y., Lin X., Chen X., Fang D., Jamieson K. PLoRa: A passive long-range data network from ambient LoRa transmissions. Proceedings of the 2018 Conference of the ACM Special Interest Group on Data Communication.

[41] Nafaa A., Taleb T., Murphy L. (2008). Forward error correction strategies for media streaming over wireless networks. IEEE Commun. Mag..

[42] Sornin N., Luis M., Eirich T., Kramp T., Hersent O. (2015). Lorawan Specification.

[43] Bertoldo S., Paredes M., Carosso L., Allegretti M., Savi P. Empirical indoor propagation models for LoRa radio link in an office environment. Proceedings of the 2019 13th European Conference on Antennas and Propagation (EuCAP).

[44] Blenn N., Kuipers F. (2017). LoRaWAN in the wild: Measurements from the things network. arXiv.

[45] Lee D.J.Y., Lee W.C.Y. Fine tune Lee model. Proceedings of the 11th IEEE International Symposium on Personal Indoor and Mobile Radio Communications (PIMRC 2000), Proceedings (Cat. No. 00TH8525).

[46] Dobrilović D., Malić M., Malić D., Sladojević S. (2017). Analyses and optimization of Lee propagation model for LoRa 868 MHz network deployments in urban areas. J. Eng. Manag. Compet. (JEMC).

[47] Paredes M., Bertoldo S., Carosso L., Lucianaz C., Marchetta E., Allegretti M., Savi P. (2019). Propagation measurements for a LoRa network in an urban environment. J. Electromagn. Waves Appl..

[48] Molisch A.F. (2012). Wireless Communications.

[49] Salous S. (2013). Radio Propagation Measurement and Channel Modelling.

[50] Casimiro A., Cecilio J., Ferreira P.M., Oliveira A., Freire P., Rodrigues M., Almeida L. (2019). AQUAMON—A Dependable Monitoring Platform based on Wireless Sensor Networks for Water Environments.

[51] Standard E. (2016). Short Range Devices (SRD) Operating in the Frequency Range 25 MHz to 1000 MHz.

[52] He R., Zhong Z., Ai B., Ding J., Guan K. (2012). Analysis of the relation between Fresnel zone and path loss exponent based on two-ray model. IEEE Antennas Wirel. Propag. Lett..

[53] Green D.B., Obaidat A. An accurate line of sight propagation performance model for ad-hoc 802.11 wireless LAN (WLAN) devices. Proceedings of the 2002 IEEE International Conference on Communications. Conference Proceedings ICC 2002 (Cat. No. 02CH37333).

[54] Atmel 42181 SAM D21. https://cdn.sparkfun.com/datasheets/Dev/Arduino/Boards/Atmel-42181-SAM-D21_Datasheet.pdf.

[55] Raspberry Pi 3 B+ Power Consumption. https://www.pidramble.com/wiki/benchmarks/power-consumption.

[56] Raspberry Pi 3 B+. https://www.raspberrypi.org/documentation/hardware/computemodule/datasheets/rpi_DATA_CM3plus_1p0.pdf.

[57] Qi J., Yang P., Newcombe L., Peng X., Yang Y., Zhao Z. (2020). An overview of data fusion techniques for Internet of Things enabled physical activity recognition and measure. Inf. Fus..

[58] Fouad M.M., Oweis N.E., Gaber T., Ahmed M., Snasel V. (2015). Data mining and fusion techniques for WSNs as a source of the big data. Procedia Comput. Sci..

